# Harms and benefits of adoptive immunotherapy for postoperative hepatocellular carcinoma: an updated review

**DOI:** 10.18632/oncotarget.14507

**Published:** 2017-01-04

**Authors:** Bao-Hong Yuan, Ru-Hong Li, Wei-Ping Yuan, Tian Yang, Tie-Jun Tong, Ning-Fu Peng, Le-Qun Li, Jian-Hong Zhong

**Affiliations:** ^1^ Department of General Surgery, YanAn Hospital Affiliated to Kunming Medical University, Kunming, P.R. China; ^2^ Department of Hepatobiliary Surgery, Affiliated Tumor Hospital of Guangxi Medical University, Nanning, P.R. China; ^3^ Department of Hepatobiliary Surgery, Eastern Hepatobiliary Surgery Hospital, Second Military Medical University, Shanghai, P.R. China; ^4^ Department of Mathematics, Hong Kong Baptist University, Hong Kong, P.R. China

**Keywords:** adjuvant, adoptive immunotherapy, hepatocellular carcinoma, meta-analysis

## Abstract

The harms and benefits of adoptive immunotherapy (AIT) for patients with postoperative hepatocellular carcinoma (HCC) are controversial among studies. This study aims to update the current evidence on efficacy and safety of AIT for patients with HCC who have received curative therapy. Electronic databases were systematically searched to identify randomized controlled trials (RCTs) and cohort studies evaluating adjuvant AIT for patients with HCC after curative therapies. Recurrence and mortality were compared between patients with or without adjuvant AIT. Eight RCTs and two cohort studies involving 2,120 patients met the eligibility criteria and were meta-analyzed. Adjuvant AIT was associated with significantly lower recurrence rate than curative therapies alone at 1 year [risk ratio (RR) 0.64, 95%CI 0.49-0.82], 3 years (RR 0.85, 95%CI 0.79-0.91) and 5 years (RR 0.90, 95%CI 0.85-0.95). Similarly, adjuvant AIT was associated with significantly lower mortality at 1 year (RR 0.64, 95%CI 0.52-0.79), 3 years (RR 0.73, 95%CI 0.65-0.81) and 5 years (RR 0.86, 95%CI 0.79-0.94). Short-term outcomes were confirmed in sensitivity analyses based on RCTs or choice of a fixed- or random-effect meta-analysis model. None of the included patients experienced grade 3 or 4 adverse events. Therefore, this update reinforces the evidence that adjuvant AIT after curative treatment for HCC lowers risk of recurrence and mortality.

## INTRODUCTION

Official guidelines [1, 2] identify hepatic resection and radiofrequency ablation (RFA) as two mainstay curative treatments for very early or early hepatocellular carcinoma (HCC). However, 5-year disease-free survival (DFS) associated with these treatments is only about 37% [3, 4]. Guidelines recommend transarterial chemoembolization (TACE) for intermediate or advanced HCC [1,2], but progression-free survival (PFS) is also unsatisfactory [5, 6]: even after more aggressive hepatic resection, the 5-year recurrence rate can be as high as 74% [7, 8]. These data indicate that even after curative surgery, patients with HCC have poor prognosis, highlighting the need for effective adjuvant therapies that improve patient outcomes.

Many postoperative or adjuvant therapies have been described for improving the prognosis of patients with HCC, including adjuvant adoptive immunotherapy (AIT) [9, 10]. Two systematic reviews from 2012 concluded that adjuvant AIT for patients with HCC after curative therapies may reduce recurrence rate but may not improve overall survival (OS) [11, 12]. These reviews included only a few small studies [13–18]. Since then, additional randomized controlled trials (RCTs) [19, 20] and cohort studies [21, 22] have been published with inconsistent findings. Therefore we wanted to perform an updated meta-analysis of the literature to gain a comprehensive understanding of the available evidence on the safety and efficacy of adjuvant AIT.

## RESULTS

### Description of studies

**Figure 1:**
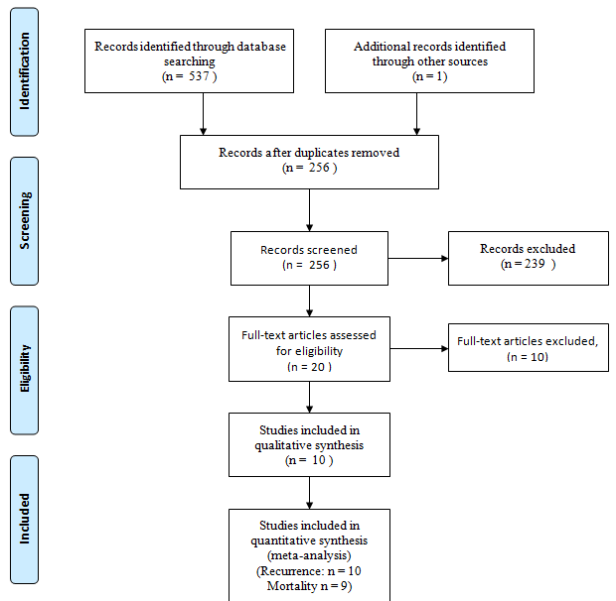
Flow chart of study selection

A total of 538 studies were identified, which decreased to 256 after duplicates were removed. Screening the titles and abstracts led to a final set of 20 studies that were read in full [13–22, 23–32]. Of these, six studies [23–28] were excluded because they contained subsets of patients already contained in larger studies [14, 15, 21, 22]. Three studies investigating AIT for patients with advanced HCC were excluded [29–31], and another study investigating a different type of postoperative immunotherapy was excluded [32]. In the end, 8 RCTs [13–20] and 2 cohort studies [21, 22] involving 1,079 AIT-treated and 1041 untreated patients were included in the meta-analysis (Figure 1, Table 1).

**Table 1:** Baseline characteristics of included studies

Study	Country	Study design	Surgery method	Child-Pugh score A/B, n (%)	Cirrhosis, *n* (%)	HBV/HCV, *n* (%)
Dong et al. 2009	China	RCT	Curative resection	102/25	101	96/NR
Huang et al. 2013	China	Retrospective	TACE+RFA	150/24 (86/14)	66	135/NR
Kawata et al. 1995	Japan	RCT	Curative resection + adriamycin	NR	14	NR
Lee, et al. 2015	Korea	RCT	Curative resection, RFA, or PEI	226/0	146	192/23
Pan et al. 2015	China	Retrospective	Curative resection	NR	NR	866/NR
Takayama et al. 2000	Japan	RCT	Curative resection	104/46	73	29/99
Weng et al. 2008	China	RCT	TACE+ RFA	69/16	NR	NR/NR
Xie et al. 2000	China	RCT	Curative resection + TACE	NR	NR	NR
Xu et al. 2016	China	RCT	Curative resection	200/0	113	171/NR
Zhou et al. 1995	China	RCT	Curative resection	NR	NR	NR/NR

Abbreviations: AIT, adoptive immunotherapy; HBV, hepatitis B virus; HCV, hepatitis C virus; NR, not reported; PEI, percutaneous ethanol injection; RCT, randomized controlled trial; RFA, radiofrequency ablation; TACE, transarterial chemoembolization.

All studies were on patient populations in Asia. Patients in two studies had undergone a sequence of TACE followed by RFA prior to AIT [16, 21]. Patients in all other studies had undergone hepatic resection prior to AIT [13–15, 17–20, 22], with patients in one trial also undergoing postoperative transarterial adriamycin chemotherapy [14], and patients in another also receiving postoperative TACE [17]. One trial [13] contained two AIT-treated arms, one treated with 3 cycles and the other with 6 cycles. Data from the two arms were combined. Of all patients in the trial by Zhou et al. [18], only those who underwent resection alone or resection followed by adjuvant AIT were included in the present meta-analysis; this trial reported recurrence data out to 1 year only [18]. Across all studies in the meta-analysis, follow-up ranged from 18 months [16] to more than 6.5 years [21] (Table 2).

**Table 2:** Study-level outcomes for HCC patients receiving adjuvant adoptive immunotherapy after curative therapies

Study	Recruitment period	Sample size (T/C)	Drugs and doses	Follow-up	Outcome and *P* value for difference ±AIT		Adverse events
**DFS or PFS**	**OS**
Dong et al. 2009	2000-2002	84/43	Group I: 3 cycles of CIK (1.0-2.0×1010); Group II: 6 cycles of CIK (1.0-2.0×1010)	>5 yr	DFS, *P* = 0.001 or 0.004*	OS, *P* = 0.884	No long-term events
Huang et al. 2013	1999-2012	85/89	NR	Median, 6.5 yr (range, 0.4-14)	PFS, *P* = 0.001	OS, *P* = 0.001	No grade 3 or 4 adverse events
Kawata et al. 1995	1989-1990	12/12	13 mg/m2 adriamycin, IL-2, and 2.5×105 LAK daily for 3 weeks	NR	DFS, *P* = 0.182	OS, *P* = 0.936	No treatment-related deaths
Lee, et al. 2015	2008-2012	114/112	16 cycles of CIK cell agent	About 3 yr	DFS, *P* = 0.01	OS, P = 0.080	No grade 3 or 4 adverse events
Pan et al. 2015	2001-2009	511/520	At least 4 cycles CIK cells (1.0-1.5×1010) via intravenous infusion	NR	PFS, *P* = 0.001	OS, *P* = 0.014	NR
Takayama et al. 2000	1992-1995	76/74	5 cycles of lymphocytes (IL-2 + Anti-CD3) (7.1×1010)	Median, 4.4 yr (range, 0.2-6.7)	DFS, *P* = 0.010	OS, *P* = 0.090	No grade 3 or 4 adverse events
Weng et al. 2008	2002-2004	45/40	39 patients received 8 cycles of CIK (1.0-1.5×1010); 6 patients received 10 cycles of CIK (1.0-1.5×1010)	Median, 1.5 yr	DFS, *P* = 0.012	100% vs. 100%	No grade 3 or 4 adverse events
Xie et al. 2000	1994-1996	21/21	TACE + transarterial injection 1×109 LAK/ IL-2 (1×106 U)	NR	DFS, *P* < 0.05	OS, *P* < 0.05	NR
Xu et al. 2016	2008-2013	100/100	4 cycles CIK cells (1.0-1.5×1010) via intravenous infusion	Median, 3.2 (range, 0.3-6.1) years	DFS, *P* = 0.334	OS, *P* = 0.141	No grade 3 or 4 adverse events
Zhou et al. 1995	1992-1992	31/30	4 cycles of LAK + IL-2	NR	DFS, *P* < 0.05	NR	NR

Abbreviations: AIT, adoptive immunotherapy; CIK, cytokine-induced killer cells; DFS, disease-free survival; IL-2, interleukin-2; LAK, lymphokine-activated killer cells; NR, not reported; OS, overall survival rate; PFS, progression-free survival; TACE, transarterial chemoembolization

* Group I or II compared to control group.

The present update substantially expands on the two previous systematic reviews comparing recurrence and mortality in patients receiving adjuvant AIT following curative therapies [11, 12]. The present work contains two RCTs [19, 20] and two cohort studies [21, 22], involving 1631 patients, that were not included in those previous reports.

### Quality of the included studies

Risks of bias in the studies in this meta-analysis were detailed in Table 3. The methodological quality was high in two studies [19, 20] (accounting for 20% of the total patient population), moderate in two [13, 15] (accounting for 13% of total patients) and low in the remaining six [14, 16, 17, 18, 21, 22] (accounting for 67% of total patients).

**Table 3:** Assessment of methodological quality (internal validity) of included studies

Study	Random allocation (description of procedure)	Concealment of random allocation	Blinding of persons who assess treatment effects	Intention-to-treat analysis
Dong et al. 2009	+	-	-	-
Huang et al. 2013	-	-	-	-
Kawata et al. 1995	-	-	-	-
Lee, et al. 2015	+	+	-	+
Pan et al. 2015	-	-	-	-
Takayama et al. 2000	+	-	-	+
Weng et al. 2008	-	-	-	-
Xie et al. 2000	-	-	-	-
Xu et al. 2016	+	+	+	+
Zhou et al. 1995	-	-	-	-

### Efficacy

Safety and efficacy data reported by each of the 10 studies in this meta-analysis [13–22] were summarized in Table 2. Eight of the 10 studies reported that adjuvant AIT significantly improved DFS or PFS (all *P* < 0.05) [13, 15–19, 21, 22], while one small RCT [17] and two retrospective studies [21, 22] reported that adjuvant AIT significantly improved OS (all *P* < 0.05).

**Figure 2:**
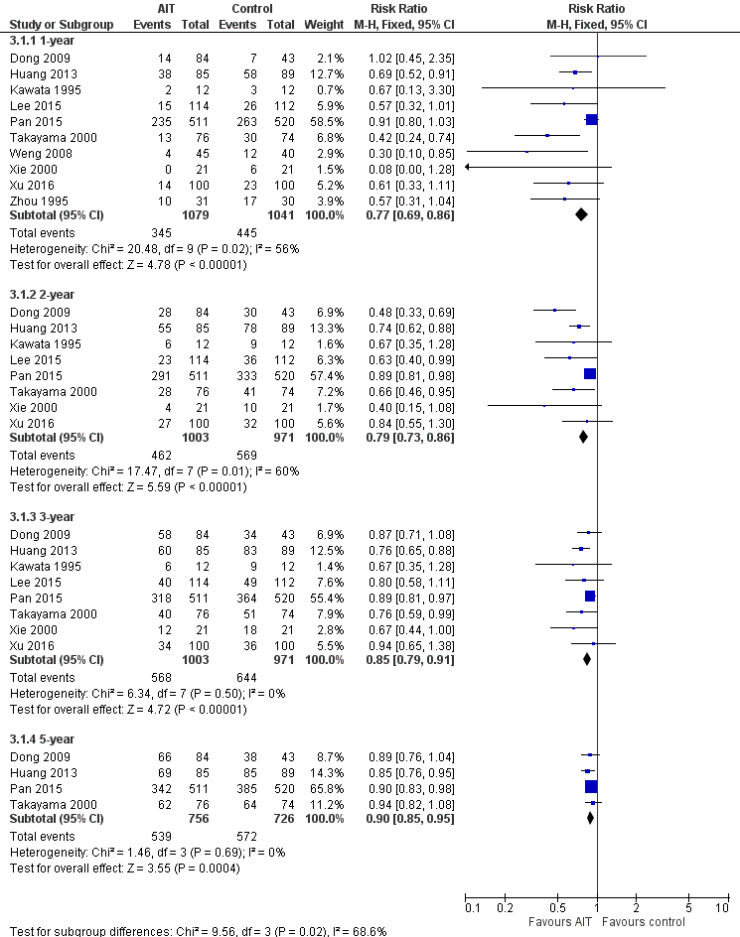
Recurrence rate of meta-analysis comparing the efficacy of adjuvant adoptive immunotherapy (AIT) with curative treatment alone

Meta-analysis of all 10 studies [13–22] suggested that adjuvant AIT was associated with significantly lower recurrence rate than curative therapies alone at 1 year (RR 0.64, 95%CI 0.49-0.82), 2 years (RR 0.70, 95%CI 0.59-0.84), 3 years (RR 0.85, 95%CI 0.79-0.91), and 5 years (RR 0.90, 95%CI 0.85-0.95) (Figure 2). Similar results were obtained using a random- or fixed-effects meta-analysis model. After excluding the two retrospective studies [21, 22], meta-analysis of the remaining 483 AIT-treated patients and 432 controls confirmed the recurrence benefit of adjuvant AIT at 1 year (RR 0.54, 95%CI 0.42-0.71), 2 years (RR 0.63, 95%CI 0.52-0.76) and 3 years (RR 0.81, 95%CI 0.71-0.93) (all *P* < 0.05). However, adjuvant AIT did not significantly reduce 5-year recurrence rate in this sensitivity analysis (RR 0.92, 95%CI 0.83-1.02).

**Figure 3:**
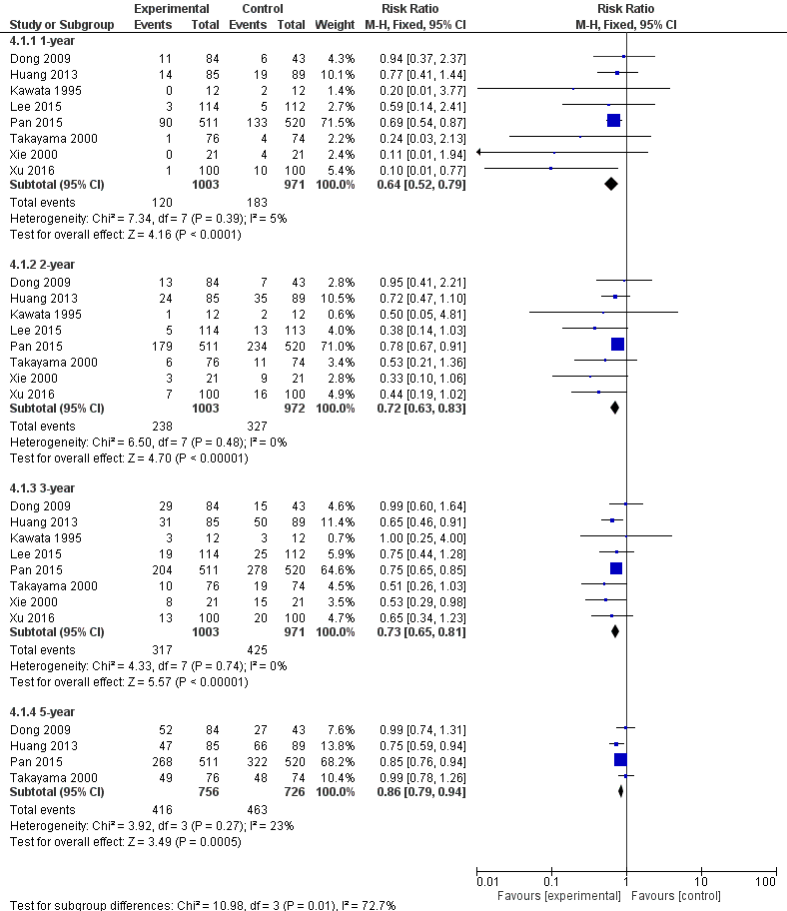
Mortality of meta-analysis comparing the efficacy of adjuvant adoptive immunotherapy (AIT) with curative treatment alone

Meta-analysis of 8 studies [13–15, 17, 19–22] suggested that adjuvant AIT was associated with significantly lower mortality than curative therapies alone at 1 year (RR 0.64, 95%CI 0.52-0.79), 2 years (RR 0.72, 95%CI 0.63-0.83), 3 years (RR 0.73, 95%CI 0.65-0.81), and 5 years (RR 0.86, 95%CI 0.79-0.94) (all *P* < 0.05; Figure 3). Similar results were obtained using a random- or fixed-effects meta-analysis model. Sensitivity analysis using data from only the 6 RCTs [13–15, 17, 19, 20] supported a benefit of adjuvant AIT for mortality at 1 year (RR 0.39, 95%CI 0.21-0.72) and 2 years (RR 0.51, 95%CI 0.34-0.76), 3 years (RR 0.71, 95%CI 0.55-0.92), but not at 5 years (RR 0.99, 95%CI 0.83-1.19).

### AIT-related adverse events

None of the 10 studies in the meta-analysis reported hospital deaths or serious adverse events attributed to adjuvant AIT. The most frequent adverse events due to AIT were grade 1 fever (defined as persistent or transient temperature of 37.5-39.3°C) and chills. The study with the highest frequency of persistent fever reported it in 5 of 84 (6.0%) patients [13], and none of the 5 was able to complete AIT per protocol because of this condition. In all patients experiencing fever in the meta-analysis, the condition was easily controlled with symptomatic therapies. Rare adverse events included headache, nausea, myalgia, fatigue, dizziness, itching, and tachycardia. All adverse events were grade 1 or 2 and self-limiting. In no case did adverse events cause patients to delay or stop treatment, except for the 5 patients with persistent fever mentioned above. No cases of infection, hepatic deterioration, pulmonary symptoms or autoimmune disorder were reported in the 10 studies.

### Assessment of publication bias

**Figure 4:**
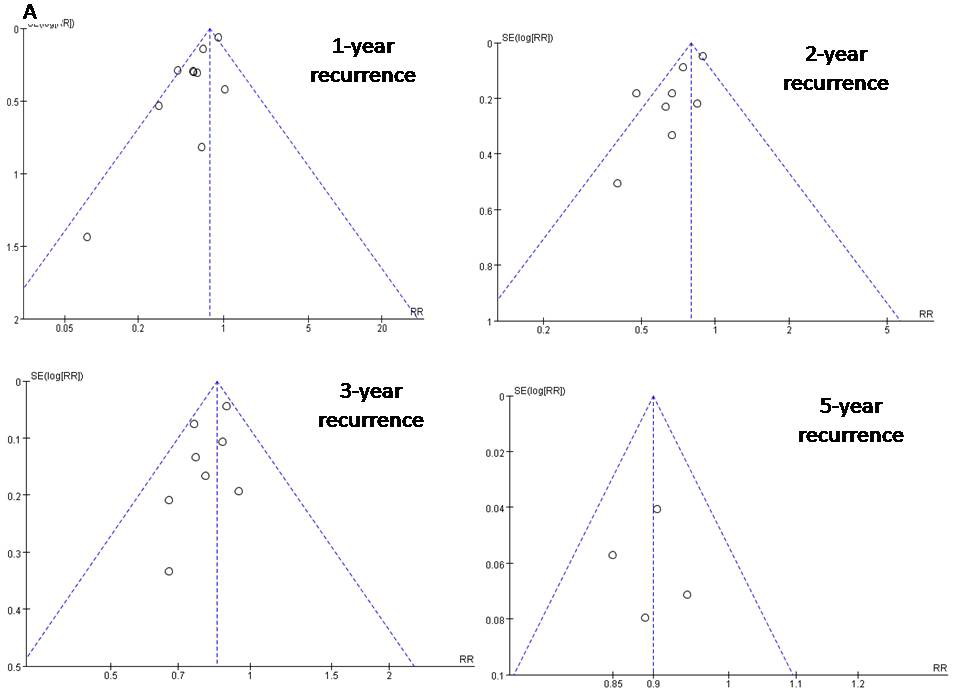
Funnel plots to detect any publication bias about recurrence rate

**Figure 5:**
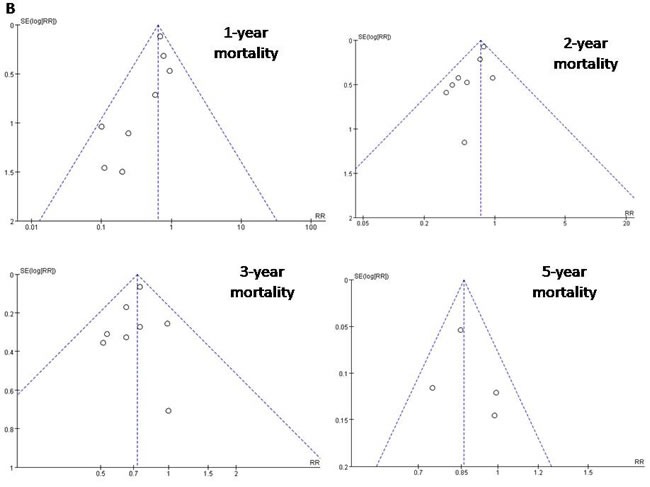
Funnel plots to detect any publication bias about mortality

Funnel plots of the 10 studies in the meta-analysis showed a symmetrical shape, suggesting minimal risk of publication bias (Figures 4 and 5).

## DISCUSSION

HCC is associated with a high recurrence rate, even after curative treatment; in fact, recurrence is the primary cause of death of all patients with HCC. Even after hepatic resection of HCC, patients with large/multinodular HCC can show 5-year DFS of 26%, while this rate can be as low as 18% in those with macrovascular invasion, based on a systematic review of more than 14,000 patients [33]. For such patients, adjuvant TACE shows promise for reducing recurrence and mortality [34]. For patients with hepatitis B virus-related HCC, postoperative antiviral therapy can be safe and effective treatment [35, 36]. However, some HCC patients are unfit for TACE or antiviral therapy after surgery. For these patients, and for those with low immune function, which is associated with HCC recurrence [37], adjuvant AIT may prevent tumor relapse. Adjuvant AIT involves transferring immune effectors into the cancer patient in the hopes of stimulating specific anti-tumor immune responses [38]. Such stimulation may counterbalance the strongly immunosuppressive microenvironment in the liver [39].

The present meta-analysis updates two systematic reviews [11, 12] from 2012 examining the safety and efficacy of adjuvant AIT for HCC patients who have received curative therapies. In contrast to those previous reports [11, 12], this update provides strong evidence that AIT can significantly reduce the rate of tumor recurrence and mortality. The discrepancy between our findings and those of previous systematic reviews likely reflects the more than two decades spanned by the literature, with the first RCTs on AIT for postoperative HCC published in 1995 [14, 18] and the most recent in 2016 [20], combined with rapid scientific and technological advances in AIT [40, 41]. In addition, no international guidelines or standards exist regarding route of AIT administration, dosing, or cycles. As a result, clinicians can vary substantially in what immune effector cells they use for AIT and what dosing/cycling protocols they follow. Indeed, in the present meta-analysis, AIT was based on three types of immunological effector cells: anti-CD3–activated peripheral blood lymphocytes, cytokine-induced killer cells, and lymphokine-activated killer cells. AIT was administered via injection into the intrahepatic artery [17, 18] or via intravenous infusion [15, 16, 19–22]. The number of cycles varied from one [17] to 16 [19]. Such heterogeneity highlights the importance of evidence updates like the present one, and the need for systematic assessment and optimization of AIT protocols, perhaps even tailored to HCC type or treatment history.

Our meta-analysis of RCT data suggests that adjuvant AIT can significantly reduce recurrence and mortality at 1, 2 and 3 years, but not 5 years. This may mean that AIT-mediated immune boosting can eliminate small intrahepatic metastases, but it does not prevent multicentric relapse in remnant liver. This hypothesis is consistent with the findings of one study [20] in our meta-analysis that reported that the ability of adjuvant AIT to prevent tumor recurrence was more obvious in the short term and less so in the long term, and that its ability to prolong time to recurrence was greater in patients with tumors >5 cm, moderately or poorly differentiated tumors, or preoperative α-fetoprotein levels ≥25 ng/mL. Though our results were supported by previous systematic reviews [42,43], the effects of adjuvant AIT on HCC recurrence in the short and long term should be investigated in greater detail.

Patients in two of the included studies underwent minimally invasive treatments [16, 21]. In one of these studies, no patient died during the 1.5-year follow-up [16]. In the other study [21], adjuvant AIT significantly improved PFS and OS. These results raise the possibility that the combination of minimally invasive treatments and postoperative AIT may exert synergistic effects. For example, since RFA is known to stimulate the differentiation of natural killer cells and boost their activity [44], it is possible that combining RFA with AIT may further boost immune function and reduce the rate of tumor recurrence. Future studies should investigate the possibility that for suitable patients, the combination of RFA and AIT may be superior to the combination of resection and AIT.

AIT may be based either on human leukocyte antigen-restricted or unrestricted strategies [45]. Cytokine-induced killer cells and lymphokine activated killer cells are heterogeneous mixture of immune effector cells that feature a mixed T- and natural killer cell-like phenotype in their terminally-differentiated CD3+CD56+ subset. Cytokine-induced killer cells and lymphokine activated killer cells can exhibit histocompatibility complex-unrestricted cytotoxicity against a broad range of tumors [46]. Transferred T-cell-based cytotoxicity is the most probable mechanism for anti-CD3-activated peripheral blood lymphocytes [15]. These natural effectors carry out their antitumoral activities without identify and recognize the presence of specific tumor associated antigens expressed on the cells surface. The easy availability, high proliferation rate and widely major histocompatibility complex-unrestricted antitumor activity of three types of cells contribute to their particularly advantageous profile, making them an attractive approach for AIT. Micrometastatic HCC cells are plausible targets. Use of peripheral blood as the source of effectors is supported by the fact that tumour-specific cytotoxic T-cells can be isolated from the peripheral repertoire. However, the extent to which specific T-cell responses contribute to the best clinical outcome needs further clinical trials.

In the past two decades, the scientific interest is focused on oral multikinase inhibitor drugs. However, adjuvant oral multikinase inhibitors provided negative efficacy for HCC after surgery, RFA, or TACE [47–51], giving the space to explore new effective adjuvant therapies.

The findings of this meta-analysis that adjuvant AIT significantly reduces recurrence and mortality for postoperative HCC must be interpreted with caution. Surgical method, type of cytokines, number of infusion cycles, and duration of maintenance AIT therapy varied among the included studies, creating substantial heterogeneity for which we could not control using sensitivity analyses. Therefore we displayed the efficacy results for each study individually in Table 2. In addition, length of follow-up varied across the studies and in some cases was too short to observe long-term efficacy of adjuvant AIT. As a result, meta-analysis of outcomes at 3 and 5 years had to be conducted on subsets of all included studies. Some studies did not clearly report procedures for randomization or allocation concealment, increasing the risk of selection or reporting bias. The fourth problem with this meta-analysis is that there are limitations in the original data, which are beyond our control, but nevertheless compromise the value of the study. We know very little about surveillance/screening methodology, diagnostic criteria for HCC, and stage systems for HCC in this meta-analysis. So, a large variability of post-treatment surveillance programs and diagnostic criteria among studies could be expected. The last relevant issue of this meta-analysis is the potential lack of external validity of the results for different populations and settings. All the included studies were conducted on patient populations in Asia. So, a high rate of hepatitis B virus infected patients with or without cirrhosis could be expected. This population may be different in terms of clinical features and comorbidities from most cases of hepatitis C virus-related or post- non-alcoholic steatohepatitis HCC from US and Europe.

Despite these limitations, our meta-analysis provides an updated picture of the evidence based on adjuvant AIT: AIT may be superior to either hepatic resection alone or the combination of TACE followed by RFA for postoperative HCC patients. The findings of the present meta-analysis should be verified and extended in further large trials with adequate follow-up. These studies should aim to expand the range of relevant endpoints examined, such as quality of life, duration of hospital stay, and cost-effectiveness. These studies should also examine the possible clinical benefits of multi-modal immune therapies.

## MATERIALS AND METHODS

### Literature search strategy

The most recent on-line versions of the following research databases were searched in June 2016 without language restrictions: Cochrane Library (http://onlinelibrary.wiley.com/cochranelibrary/search), Wiley Online Library, Science Direct, Web of Science, Chinese National Knowledge Infrastructure, Embase, and PubMed. The following search terms were used to identify comparative studies: ‘hepatocellular carcinoma’ *or* HCC *or* ‘hepatic cancer’ *or* ‘hepatic tumor’ *or* ‘liver tumor’ *or* ‘liver cancer’, *and* ‘hepatic resection’ *or* hepatectomy *or* ‘liver resection’ *or* ‘transarterial chemoembolization’ *or* ‘radiofrequency ablation’ *or* ‘invasive treatment’ *or* ‘percutaneous ethanol infection’, *and* ‘adoptive immunotherapy’ *or* ‘cytokine induced killer cells’ *or* ‘lymphokine activated killer cells’ *or* ‘tumor infiltrating lymphocytes’ *or* ‘interleukin-2’. To avoid missing relevant studies, no filter was imposed to exclude non-controlled studies or non-RCTs. Relevant references were also searched manually to identify additional studies.

### Inclusion criteria

We included in the meta-analysis full-length research studies that satisfied the following criteria: (a) the study compared the efficacy of curative therapies with or without adjuvant AIT for patients with HCC; (b) the study had a randomized control or cohort design; (c) all tumors were treated by curative procedures before AIT; (d) patients in the AIT and no-AIT arms received otherwise similar treatments; and (e) the study reported sufficient data for estimating risk ratios (RRs) with 95% confidence intervals (95%CIs). Curative therapies for HCC included hepatic resection, RFA, percutaneous ethanol injection, and liver transplantation; these therapies were considered curative if no residual tumor was observed one month after the initial therapy. TACE was defined as palliative therapy for the purposes of this meta-analysis.

Studies were excluded if they evaluated the efficacy of AIT for patients with liver metastases or with recurrent, advanced, or unresectable HCC. Conference abstracts and other forms of summary publication were also excluded. In the case of multiple studies apparently based on the same population, we included only the study with the largest number of participants.

### Study identification and data extraction

Studies identified in literature searches were independently screened by two authors (B.-H.Y, R.-H.L), with discrepancies arbitrated by a third author (J.-H.Z). Two authors (B.-H.Y, R.-H.L) independently extracted the following data from included studies using a predefined template: author details, country, study design, surgery method, liver disease, recruitment period, sample size, follow-up period, interventions (drugs, schedules and numbers of therapy sessions), outcomes (positive and negative findings), and methodological quality. A third author (J.-H.Z) checked the extracted data against the original studies. Survival data were taken directly from tables or the text whenever possible; if such data were presented only in graphs, they were extracted by manual interpolation [33]. *P* values associated with inter-group differences in PFS, DFS, or OS were extracted directly from survival curves, text, or tables wherever possible.

### Outcome measures

The primary outcomes in this meta-analysis were recurrence rate and mortality. The secondary outcome was treatment-related adverse events, which included treatment-related withdrawals and discontinuations.

### Quality assessment

This meta-analysis was conducted in accordance with the Quality of Reporting of Meta-analyses (QUOROM) statement [52]. Two authors (B.-H.Y, R.-H.L) independently evaluated all included RCTs based on method of randomization, allocation concealment, blinding of outcome assessors, and use of intention-to-treat analysis. RCTs were considered to be of low quality if they reported none of the items, of moderate quality if they reported on fewer than three items and of high quality if they reported on three or four items [12, 52]. Quasi-randomized studies and cohort studies were defined to be of low quality.

### Missing data

Meta-analysis was performed on an intention-to-treat basis. To assess attrition bias, we calculated recurrence and mortality using a ‘worst-case’ approach in which patients with missing data were counted as treatment failures (recurrence or death). For patients with missing data, we ‘carried forward’ data from the most recent measurement.

### Statistical analysis

Review Manager 5.3 (Cochrane Collaboration) was used to analyze data from included studies. Due to the high likelihood of recurrence and mortality, RRs with corresponding 95%CIs were calculated for dichotomous outcomes using the Mantel-Haenszel method. Point estimates of RR were considered statistically significant when *P* < 0.05. Meta-analysis was carried out using a random-effects model if substantial heterogeneity according to an I-squared threshold was found among included studies; otherwise, the analysis was carried out using a fixed-effect model [53]. If the two models gave different results, we reported both results. Heterogeneity was assessed by calculating I^2^. Homogeneity between studies was analyzed using the χ^2^ test, with significance set at *P* > 0.1. Publication bias was assessed by visual inspection of Begg's funnel plots. Sensitivity analyses excluding cohort studies and choice of random- or fixed-effect meta-analysis model were performed.

## References

[1] National Comprehensive Cancer Network (NCCN) (2016). NCCN Clinical Practice Guidelines in Oncology. Hepatobiliary Cancers.

[2] European Association For The Study Of The Liver; European Organisation For Research And Treatment Of Cancer. EASL-EORTC clinical practice guidelines: management of hepatocellular carcinoma. J Hepatol.

[3] Lim KC, Chow PK, Allen JC, Siddiqui FJ, Chan ES, Tan SB Systematic review of outcomes of liver resection for early hepatocellular carcinoma within the Milan criteria. Br J Surg.

[4] Li W, You X, Li L, Zhong J [Hepatic resection for hepatocellular carcinoma involving a single large tumor, multiple tumors or macrovascular invasion]. Zhonghua Yi Xue Za Zhi.

[5] Zhang T, Zhao YT, Wang Z, Li CR, Jin J, Jia AY, Wang SL, Song YW, Liu YP, Ren H, Fang H, Bao H Efficacy and Safety of Intensity-Modulated Radiotherapy Following Transarterial Chemoembolization in Patients With Unresectable Hepatocellular Carcinoma. Medicine (Baltimore).

[6] Si ZM, Wang GZ, Qian S, Qu XD, Yan ZP, Liu R, Wang JH Combination Therapies in the Management of Large (≥ 5 cm) Hepatocellular Carcinoma: Microwave Ablation Immediately Followed by Transarterial Chemoembolization. J Vasc Interv Radiol.

[7] Zhong JH, Ke Y, Gong WF, Xiang BD, Ma L, Ye XP, Peng T, Xie GS, Li LQ Hepatic resection associated with good survival for selected patients with intermediate and advanced-stage hepatocellular carcinoma. Ann Surg.

[8] Torzilli G, Belghiti J, Kokudo N, Takayama T, Capussotti L, Nuzzo G, Vauthey JN, Choti MA, De Santibanes E, Donadon M, Morenghi E, Makuuchi M A snapshot of the effective indications and results of surgery for hepatocellular carcinoma in tertiary referral centers: is it adherent to the EASL/AASLD recommendations?: an observational study of the HCC East-West study group. Ann Surg.

[9] Zhong JH, Ma L, Li LQ Postoperative therapy options for hepatocellular carcinoma. Scand J Gastroenterol.

[10] Zhu GQ, Shi KQ, Yu HJ, He SY, Braddock M, Zhou MT, Chen YP, Zheng MH Optimal adjuvant therapy for resected hepatocellular carcinoma: a systematic review with network meta-analysis. Oncotarget.

[11] Xie F, Zhang X, Li H, Zheng T, Xu F, Shen R, Yan L, Yang J, He J Adoptive immunotherapy in postoperative hepatocellular carcinoma: a systemic review. PLoS One.

[12] Zhong JH, Ma L, Wu LC, Zhao W, Yuan WP, Wu FX, Zhang ZM, Huang S, You XM, Li LQ Adoptive immunotherapy for postoperative hepatocellular carcinoma: a systematic review. Int J Clin Pract.

[13] Hui D, Qiang L, Jian W, Ti Z, Da-Lu K A randomized, controlled trial of postoperative adjuvant cytokine-induced killer cells immunotherapy after radical resection of hepatocellular carcinoma. Dig Liver Dis.

[14] Kawata A, Une Y, Hosokawa M, Wakizaka Y, Namieno T, Uchino J, Kobayashi H Adjuvant chemoimmunotherapy for hepatocellular carcinoma patients. Adriamycin, interleukin-2, and lymphokine-activated killer cells versus adriamycin alone. Am J Clin Oncol.

[15] Takayama T, Sekine T, Makuuchi M, Yamasaki S, Kosuge T, Yamamoto J, Shimada K, Sakamoto M, Hirohashi S, Ohashi Y, Kakizoe T Adoptive immunotherapy to lower postsurgical recurrence rates of hepatocellular carcinoma: a randomised trial. Lancet.

[16] Weng DS, Zhou J, Zhou QM, Zhao M, Wang QJ, Huang LX, Li YQ, Chen SP, Wu PH, Xia JC Minimally invasive treatment combined with cytokine-induced killer cells therapy lower the short-term recurrence rates of hepatocellular carcinomas. J Immunother.

[17] Xie L, Pang R, Jin Y, Xiang S, Li H [Effects of hepatic artery chemotherapeutic embolization combined with perfusing LAK cells into hepatic artery after radical operation of liver cancer]. Zhonghua Gan Zang Bing Za Zhi.

[18] Zhou WP, Wu MC, Chen H [The effects of combined hepatectomy and immuno-chemotherapy on postoperative recurrence rate of primary liver cancer]. Zhonghua Wai Ke Za Zhi.

[19] Lee JH, Lim YS, Yeon JE, Yeon JE, Song TJ, Yu SJ, Gwak GY, Kim KM, Kim YJ, Lee JW, Yoon JH Adjuvant immunotherapy with autologous cytokine-induced killer cells for hepatocellular carcinoma. Gastroenterology.

[20] Xu L, Wang J, Kim Y, Shuang ZY, Zhang YJ, Lao XM, Li YQ, Chen MS, Pawlik TM, Xia JC, Li SP, Lau WY A randomized controlled trial on patients with or without adjuvant autologous cytokine-induced killer cells after curative resection for hepatocellular carcinoma. Oncoimmunology.

[21] Huang ZM, Li W, Li S, Gao F, Zhou QM, Wu FM, He N, Pan CC, Xia JC, Wu PH, Zhao M Cytokine-induced killer cells in combination with transcatheter arterial chemoembolization and radiofrequency ablation for hepatocellular carcinoma patients. J Immunother.

[22] Pan QZ, Wang QJ, Dan JQ, Pan K, Li YQ, Zhang YJ, Zhao JJ, Weng DS, Tang Y, Huang LX, He J, Chen SP, Ke ML A nomogram for predicting the benefit of adjuvant cytokine-induced killer cell immunotherapy in patients with hepatocellular carcinoma. Sci Rep.

[23] Chen JL, Lao XM, Lin XJ, Xu L, Cui BK, Wang J, Lin GH, Shuang ZY, Mao YZ, Huang X, Yun JP, Jin JT, Li SP Adjuvant Cytokine-Induced Killer Cell Therapy Improves Disease-Free and Overall Survival in Solitary and Nonmicrovascular Invasive Hepatocellular Carcinoma After Curative Resection. Medicine (Baltimore).

[24] Pan CC, Huang ZL, Li W, Zhao M, Zhou QM, Xia JC, Wu PH Serum alpha-fetoprotein measurement in predicting clinical outcome related to autologous cytokine-induced killer cells in patients with hepatocellular carcinoma undergone minimally invasive therapy. Chin J Cancer.

[25] Pan K, Li YQ, Wang W, Xu L, Zhang YJ, Zheng HX, Zhao JJ, Qiu HJ, Weng DS, Li JJ, Wang QJ, Huang LX, He J The efficacy of cytokine-induced killer cell infusion as an adjuvant therapy for postoperative hepatocellular carcinoma patients. Ann Surg Oncol.

[26] Takayama T, Makuuchi M Prevention of hepatocellular carcinoma recurrence: actuality and perspectives. Hepatogastroenterology.

[27] Uchino J, Une Y, Kawata A, Wakisaka Y, Hosokawa M Postoperative chemoimmunotherapy for the treatment of liver cancer. Semin Surg Oncol.

[28] Zhou QM, Wu PH, Zhao M, Wang QJ, Huang LX, Li YQ, Chen SP, Xia JC [Short-term curative efficacy of cytokine-induced killer cells combined micro-invasive treatments on hepatocellular carcinoma]. Ai Zheng.

[29] Guo W, Liu L, Wu D [Dendritic cell-cytokine induced killer cell immunotherapy combined with transcatheter arterial chemoembolization for hepatocellular carcinoma: safety and efficacy]. Nan Fang Yi Ke Da Xue Xue Bao.

[30] Hao MZ, Lin HL, Chen Q, Ye YB, Chen QZ, Chen MS Efficacy of transcatheter arterial chemoembolization combined with cytokine-induced killer cell therapy on hepatocellular carcinoma: a comparative study. Chin J Cancer.

[31] Yu X, Zhao H, Liu L, Cao S, Ren B, Zhang N, An X, Yu J, Li H, Ren X A randomized phase II study of autologous cytokine-induced killer cells in treatment of hepatocellular carcinoma. J Clin Immunol.

[32] Peng B, Liang L, Chen Z, He Q, Kuang M, Zhou F, Lu M, Huang J Autologous tumor vaccine lowering postsurgical recurrent rate of hepatocellular carcinoma. Hepatogastroenterology.

[33] Zhong JH, Rodriguez AC, Ke Y, Wang YY, Wang L, Li LQ Hepatic resection as a safe and effective treatment for hepatocellular carcinoma involving a single large tumor, multiple tumors, or macrovascular invasion. Medicine (Baltimore).

[34] Zhong JH, Li LQ Postoperative adjuvant transarterial chemoembolization for participants with hepatocellular carcinoma: A meta-analysis. Hepatol Res.

[35] Huang G, Lau WY, Wang ZG, Pan ZY, Yuan SX, Shen F, Zhou WP, Wu MC Antiviral therapy improves postoperative survival in patients with hepatocellular carcinoma: a randomized controlled trial. Ann Surg.

[36] Ke Y, Ma L, You XM, Huang SX, Liang YR, Xiang BD, Li LQ, Zhong JH Antiviral therapy for hepatitis B virus-related hepatocellular carcinoma after radical hepatectomy. Cancer Biol Med.

[37] Cheng JW, Shi YH, Fan J, Huang XW, Qiu SJ, Xiao YS, Wang Z, Dai Z, Tang ZY, Zhou J An immune function assay predicts post-transplant recurrence in patients with hepatocellular carcinoma. J Cancer Res Clin Oncol.

[38] Morse MA, Clay TM, Lyerly HK Current status of adoptive immunotherapy of malignancies. Expert Opin Biol Ther.

[39] Tagliamonte M, Petrizzo A, Tornesello ML, Ciliberto G, Buonaguro FM, Buonaguro L Combinatorial immunotherapy strategies for hepatocellular carcinoma. Curr Opin Immunol.

[40] Prieto J, Melero I, Sangro B Immunological landscape and immunotherapy of hepatocellular carcinoma. Nat Rev Gastroenterol Hepatol.

[41] Harding JJ, El Dika I, Abou-Alfa GK Immunotherapy in hepatocellular carcinoma: Primed to make a difference?. Cancer.

[42] Li YC, Zhao L, Wu JP, Qu CX, Song QK, Wang RB Cytokine-induced killer cell infusion combined with conventional treatments produced better prognosis for hepatocellular carcinoma patients with barcelona clinic liver cancer B or earlier stage: A systematic review and meta-analysis. Cytotherapy.

[43] Wang H, Liu A, Bo W, Feng X, Hu Y, Tian L, Zhang H, Tang X Adjuvant immunotherapy with autologous cytokine-induced killer cells for hepatocellular carcinoma patients after curative resection, a systematic review and meta-analysis. Dig Liver Dis.

[44] Zerbini A, Pilli M, Laccabue D, Pelosi G, Molinari A, Negri E, Cerioni S, Fagnoni F, Soliani P, Ferrari C, Missale G Radiofrequency thermal ablation for hepatocellular carcinoma stimulates autologous NK-cell response. Gastroenterology.

[45] Rosenberg SA, Restifo NP Adoptive cell transfer as personalized immunotherapy for human cancer. Science.

[46] Schmeel FC, Schmeel LC, Gast SM, Schmidt-Wolf IG Adoptive immunotherapy strategies with cytokine-induced killer (CIK) cells in the treatment of hematological malignancies. Int J Mol Sci.

[47] Zhong JH The STORM trial and beyond: narrowing the horizon of adjuvant sorafenib for postoperative hepatocellular carcinoma. Tumour Biol.

[48] Bruix J, Takayama T, Mazzaferro V, Chau GY, Yang J, Kudo M, Cai J, Poon RT, Han KH, Tak WY, Lee HC, Song T, Roayaie S Adjuvant sorafenib for hepatocellular carcinoma after resection or ablation (STORM): a phase 3, randomised, double-blind, placebo-controlled trial. Lancet Oncol.

[49] Kudo M, Han G, Finn RS, Poon RT, Blanc JF, Yan L, Yang J, Lu L, Tak WY, Yu X, Lee JH, Lin SM, Wu C Brivanib as adjuvant therapy to transarterial chemoembolization in patients with hepatocellular carcinoma: A randomized phase III trial. Hepatology.

[50] Kudo M, Imanaka K, Chida N, Nakachi K, Tak WY, Takayama T, Yoon JH, Hori T, Kumada H, Hayashi N, Kaneko S, Tsubouchi H, Suh DJ Phase III study of sorafenib after transarterial chemoembolisation in Japanese and Korean patients with unresectable hepatocellular carcinoma. Eur J Cancer.

[51] Zhong JH, Du XK, Xiang BD, Li LQ Adjuvant sorafenib in hepatocellular carcinoma: A cautionary comment of STORM trial. World J Hepatol.

[52] Moher D, Cook DJ, Eastwood S, Olkin I, Rennie D, Stroup DF Improving the quality of reports of meta-analyses of randomised controlled trials: the QUOROM statement. Quality of Reporting of Meta-analyses. Lancet.

[53] Borenstein M, Hedges LV, Higgins JP, Rothstein HR A basic introduction to fixed-effect and random-effects models for meta-analysis. Res Synth Methods.

